# Case Report: Anti-neutrophil Cytoplasmic Antibody-Associated Vasculitis With Acute Renal Failure and Pulmonary Hemorrhage May Occur After COVID-19 Vaccination

**DOI:** 10.3389/fmed.2021.765447

**Published:** 2021-11-11

**Authors:** Chien-Chou Chen, Hsin-Yang Chen, Chun-Chi Lu, Shih-Hua Lin

**Affiliations:** ^1^Department of Internal Medicine, Tri-service General Hospital Songshan Branch, National Defense Medical Center, Taipei, Taiwan; ^2^Division of Nephrology, Department of Medicine, Tri-service General Hospital, National Defense Medical Center, Taipei, Taiwan; ^3^Division of Rheumatology, Department of Medicine, Tri-service General Hospital, National Defense Medical Center, Taipei, Taiwan

**Keywords:** anti-neutrophil cytoplasmic antibody (ANCA), vasculitis, vaccination, COVID-19, pulmonary renal syndrome

## Abstract

The rare and severe adverse effects associated with coronavirus disease of 2019 (COVID-19) vaccination have been under-appreciated, resulting in many instances of inappropriate management. We describe the case of an elderly woman who developed anti-neutrophil cytoplasmic antibody-associated vasculitis with pulmonary renal syndrome approximately 3 weeks after the first dose of COVID-19 mRNA vaccination (Moderna). Her nasopharyngeal polymerase chain reaction test for the COVID-19 RNA virus was negative. Gross hematuria, heavy proteinuria, acute renal failure (serum creatinine up to 6.5 mg/dL), and hemoptysis coupled with a marked increase in serum anti-myeloperoxidase-O antibody were observed. Renal biopsy showed severe vasculitis with pauci-immune crescent glomerulonephritis. The pulmonary hemorrhage was resolved and renal function improved following combined plasma exchange and the administration of systemic steroids and anti-CD20 therapy. The early examination of urinalysis and renal function may be crucial for identifying glomerulonephritis and acute renal failure in susceptible patients after COVID-19 vaccination.

## Introduction

The global pandemic caused by severe acute respiratory syndrome coronavirus 2 has led to a significant loss of life, as well as severe disruptions to economies and social activities worldwide. Coronavirus disease of 2019 (COVID-19) infection will progress to acute respiratory distress syndrome in approximately 15–30% of hospitalized patients (1). To date, over 214 million COVID-19 cases and 4 million COVID-19-related deaths have been reported globally (2). COVID-19 vaccines have been rapidly developed, and their effectiveness and safety have been demonstrated in clinical trials. The prompt implementation of mass vaccination in a large number of countries has greatly reduced COVID-19-related mortality (3). Current types of COVID-19 vaccines include the following: mRNA delivered via lipid nanoparticles, viral vectors, inactivated virus, and protein subunit vaccines (3).

Due to the enhancement of the immune response by COVID-19 vaccinations, rare and serious adverse effects have been recently reported. These include vaccine-induced immune thrombotic thrombocytopenia and immune-mediated myocarditis associated with the use of viral vector vaccines and mRNA vaccines, respectively (4, 5). An increasing number of studies has also supported a possible link between COVID-19 vaccination and the rapid development of *de novo* or relapsed glomerular diseases, such as anti-neutrophil cytoplasmic antibody-associated vasculitis (AAV). AAV is an autoimmune disorder characterized by small vascular inflammation, which predominantly occurs in the kidneys and in the presence of anti-neutrophil cytoplasmic antibodies and either anti-myeloperoxidase or anti-proteinase 3 antibodies (6, 7). Here, we describe an elderly patient who developed life-threatening AAV and rapidly progressive glomerulonephritis (RPGN) and pulmonary hemorrhage 3 weeks after her first COVID-19 mRNA vaccination. The patient was successfully treated with plasma exchange and the administration of systemic steroids and anti-CD20 therapy.

## Case Report

A 70-year-old healthy woman was hospitalized due to worsening hematuria, proteinuria, and acute renal failure. She did not have a history of prior COVID-19 infection or medical illness (with the exception of urinary tract infection, from which she had recovered in March). The patient reported that she experienced dizziness, headache, and hematuria 1 week after the first dose of mRNA-1273 COVID-19 vaccination (Moderna); this was followed by a progressive elevation of blood pressure, gross hematuria, and decreased urine levels 2 weeks later. She was diagnosed with acute renal failure (with an incremental serum creatinine level ranging from 1.0 to 3.5 mg/dL) at a district hospital and were subsequently referred to medical center.

The patient was alert and oriented on admission, but developed hemoptysis and exertional dyspnea. Her supine blood pressure, heart rate, and body temperature were 160/90 mmHg, 88 beats/min, and 37.8°C, respectively. Pulse oximetry indicated that her blood oxygen saturation was 96% with 28% oxygen support. The following features were noted: pale conjunctiva, shallow breathing, rhonchi, rales over the left lung field, and bilateral leg edema. The rest of her physical examination was unremarkable. Two nasopharyngeal swabs were obtained for the COVID-19 polymerase chain reaction test, both yielding negative results. Urinalysis revealed more than 100 red blood cells per high-power field and nephrotic-range proteinuria (urine protein creatinine ratio, 4,384 mg/g). Abnormal laboratory test values included hemoglobin (8.7 g/dL), creatinine (3.5 mg/dL), blood urea nitrogen (88 mg/dL), albumin (3.1 g/dL), and C-reactive protein (2.7 mg/dL) levels. Arterial blood gas analysis showed pH, partial pressure of carbon dioxide, partial pressure of oxygen, and HCO_3_ values of 7.45, 26.1 mmHg, 86.3 mmHg, and 17.5 mmol/L, respectively.

**Figure 1:**
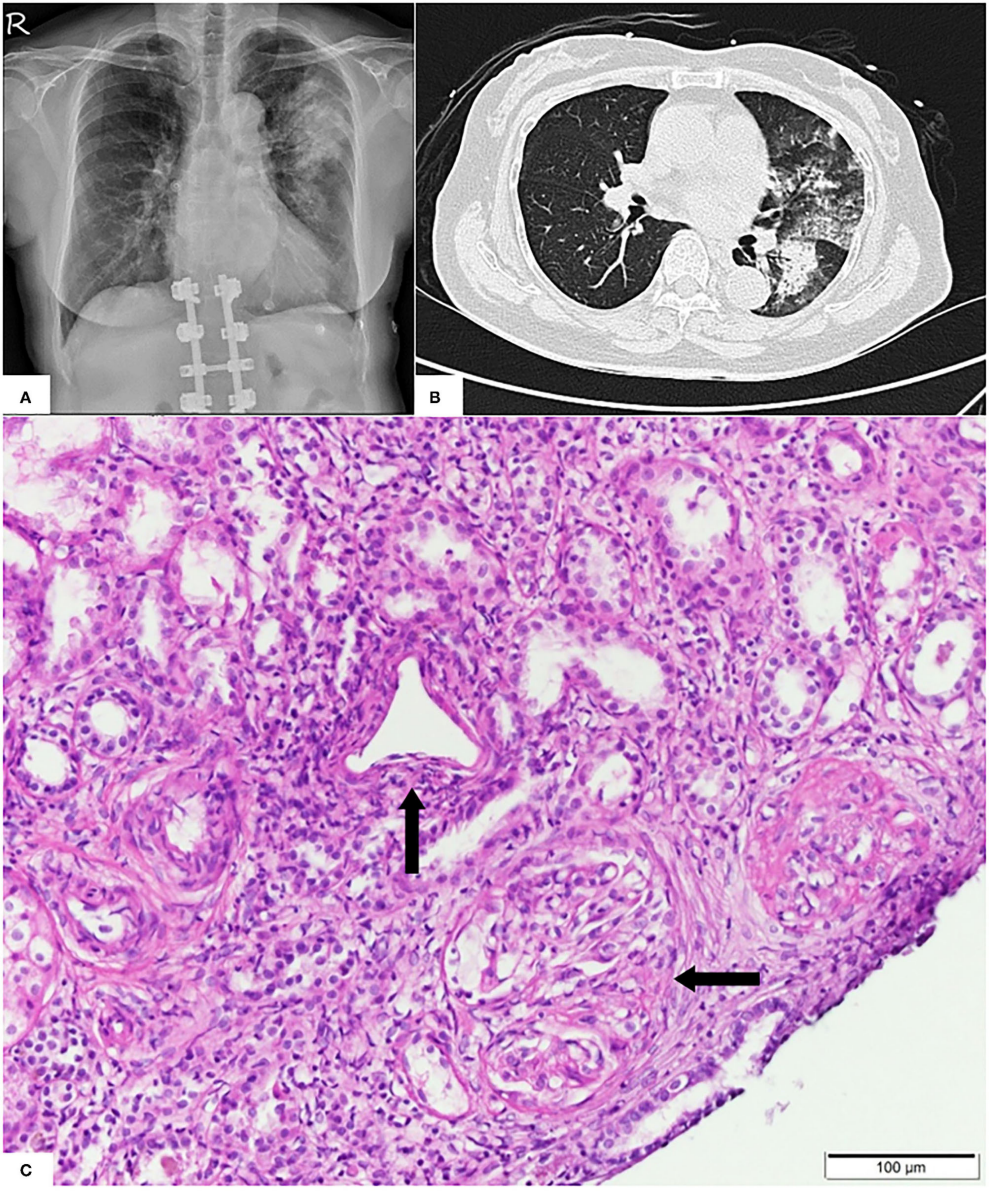
**(A)** Pulmonary X-ray showing dense patchy consolidations in the left upper lung field. **(B)** Pulmonary computed tomography revealing peribronchial consolidations and ground glass opacities in the bilateral lung field, predominantly in the left upper lobe. **(C)** Light microscopy showing predominant vasculitis (parallel arrow) with pauci-immune crescent glomerulonephritis (horizontal arrow).

Pulmonary radiography and computed tomography showed bilateral peribronchial consolidations, which were predominantly located in the left upper lung (Figures 1A,B). Echocardiography and abdominal ultrasonography yielded normal findings. The results of immediate and thorough examinations (including immune, infection, and tumor surveys) for RPGN were unremarkable, except for a notable increase in anti-myeloperoxidase-O antibodies (470 IU/mL; the normal level is <5 IU/L). After one session of emergent plasma exchange and intravenous steroid therapy, kidney biopsy was performed after obtaining informed consent, demonstrating acute severe renal vasculitis with pauci-immune crescent glomerulonephritis (Figure 1C).

**Figure 2:**
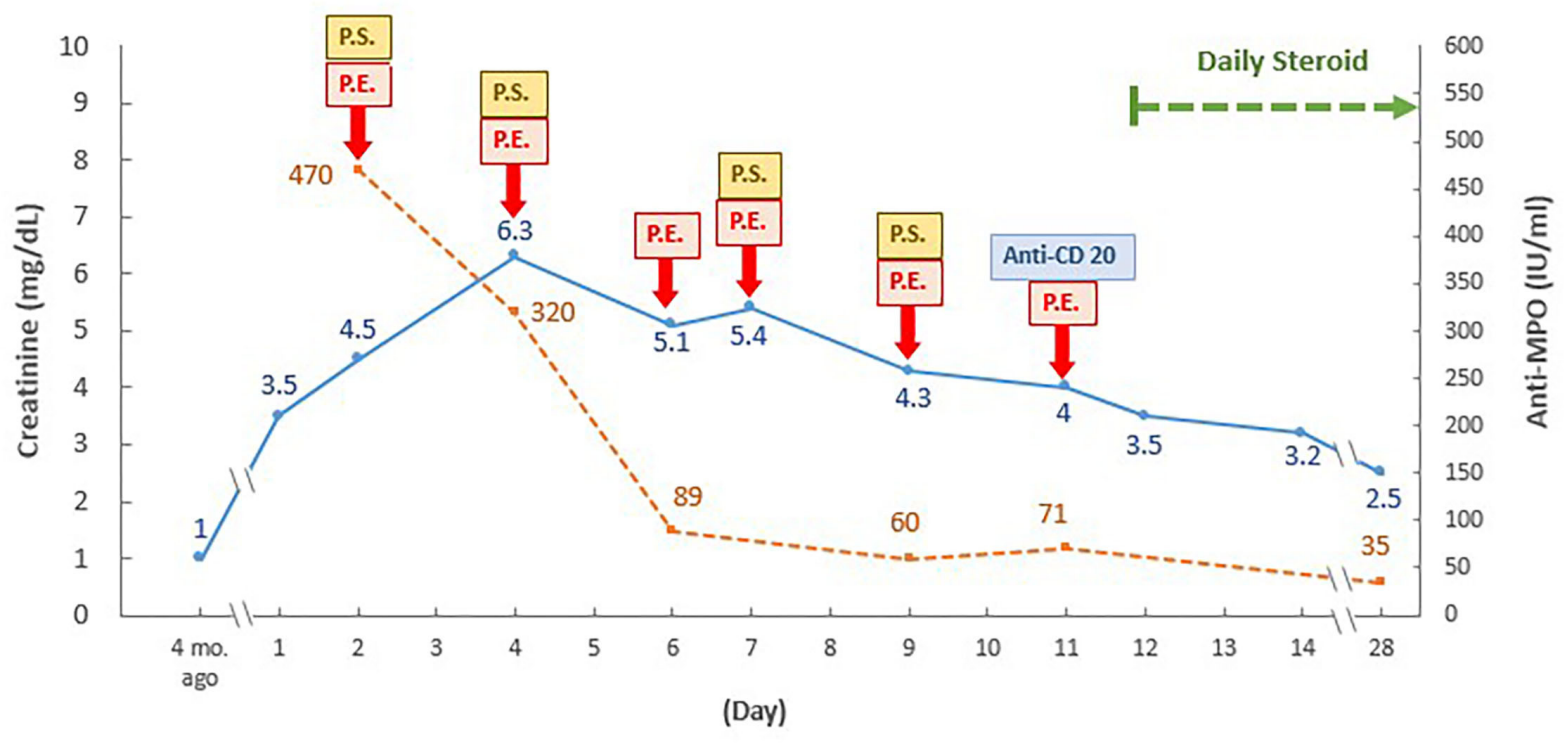
Time and therapeutic course of anti-neutrophil cytoplasmic antibody-associated vasculitis with rapidly progressive glomerulonephritis following COVID-19 vaccination. PE, plasma exchange; P.S., pulse steroid; ANCA, anti-neutrophil cytoplasmic antibody; Anti-MPO, anti-myeloperoxidase-O, mo., month. Daily pulse steroid dosage: intravenous methylprednisolone 1,000 mg daily in the first course, then 500 mg daily in the following three courses.

The pulmonary hemorrhage was resolved and renal function was improved (serum creatinine, 2.5 mg/dL) 3 weeks later, following the continuous administration of combined plasma exchange, systemic steroid, and anti-CD20 therapy (500 mg) (Figure 2). While the patient still exhibited persistent microscopic hematuria (10–75 red blood cells per high-power field), proteinuria (<1.0 g/day) was attenuated via daily steroid treatment (25 mg/day).

## Discussion

The patient in the present case showed typical features of AAV with RPGN and pulmonary involvement during the COVID-19 pandemic. While patients with COVID-19 infection may exhibit coexisting pneumonia and acute renal failure, thus mimicking pulmonary renal syndrome, this possibility was excluded by two negative COVID-19 polymerase chain reaction tests. As there were no other identifiable causes (e.g., other infections, malignancies, or drugs) of AAV, the etiological factor was concluded to be the patient's first dose of the COVID-19 mRNA vaccine.

To date, eight cases (including the present report) have shown temporal links between the development of *de novo* AAV with RPGN and COVID-19 vaccination (Table 1). Prior cases have been mainly reported among elderly individuals without known renal diseases. Six cases have been associated with a mRNA vaccine, while a viral vector vaccine has been associated with two cases (8–14). AAV was diagnosed after either the first dose (37.5%) or second dose (62.5%) of COVID-19 vaccination. The onset time of symptoms varied from <1 week to 7 weeks (8–14). While non-specific prodromal symptoms were observed after vaccination, characteristic findings included microscopic or gross hematuria with dysmorphic red blood cells, significant proteinuria, and acute renal failure (8–14). Serum creatinine levels ranged from 1.9 to 8.4 mg/dL (8, 10–14). Extra-renal involvement with pulmonary hemorrhage, necrotic masses, consolidations and rhabdomyolysis were observed in four cases (9, 13, 14). Although a range of different therapeutic treatments were reported, most patients exhibited improvements in renal function and pulmonary symptoms; however, one patient (serum creatinine level, 8.4 mg/dL) required dialysis due to irreversible renal damage (Table 1).

**Table 1:** AAV with RPGN following COVID-19 vaccination.

**No**	**Age/Sex**	**Past Hx**	**COVID-19 vaccination**	**Onset**	**Symptoms**	**Urinalysis**	**Blood test**	**ANCA type**	**Kidney Bx**	**Extra-renal involvement**	**Treatment**	**Outcome**
1^8^	52/M	HTN	Moderna	2 weeks after 2nd dose	Headache Weakness	Dysmorphic RBC	SCr:8.4 mg/dL,	Anti-PR3 (+)	Pauci-immune necrotizing and crescentic GN	None	Hemodialysis Rituximab CYC	Dialysis-dependent
2^9^	81/M	Healthy	Moderna	Shortly after 2nd dose	Flu-like symptoms	NA	AKI	Anti-PR3 (+)	Pauci-immune crescentic GN with capillary necrosis & vasculitis	Pulmonary necrotic masses	Plasma exchange Pulse steroid CYC	Improved renal function
3^10^	77/M	Healthy	AstraZeneca	4 weeks after 1st dose	Fever, Night sweating, Anorexia	NA	SCr:2.7 mg/dL, CRP: 20 mg/dL	NA	Non-caseating, non-necrotizing granuloma	None	Pulse steroid	Resolved renal function
4^11^	78/F	DM, HTN	Pfizer-BioNTech	Immediate after 2nd dose	Nausea, vomiting, diarrhea, lethargy	Dysmorphic RBC UACR:2,050 mg/g	SCr:3.5 mg/dL,	Anti-MPO (+)	Necrotizing crescentic GN	None	Rituximab	Improved renal function(SCr: 1.7mg/dL)
5^12^	29/F	Congenital cystic lung disease	Pfizer-BioNTech	7 weeks after 2nd dose	NA	HematuriaUACR:633 mg/g	SCr: 1.9 mg/dL	Anti-MPO (71 AU/ml)	Pauci-immune crescent GN	Chronic lung infiltration	Pulse steroid Rituximab CYC	Improved renal function(SCr: 1.0 mg/dL)
6^13^	63/M	Healthy	AstraZeneca	1 week after 1st dose	Hemoptysis Flu-like symptoms	Microscopic hematuria	SCr:2.9 mg/dL	Anti-MPO (12 IU/ml)	Focal class of pauci-immune crescent GN	Infiltration over LLL	Pulse steroid	Improved renal function(SCr: 2.1 mg/dL)
7^14^	79/F	HTN	Pfizer-BioNTech	2 weeks after 2nd dose	Weakness Upper thigh pain	HematuriaUPCR: >18,000 mg/gUACR <5,000 mg/g	SCr: 6.57 mg/dL Eosinophils: 23.3%	Anti-MPO (>134 IU/ml)	Vasculitis with pauci-immune crescent GN	CK: 14,243 U/LMyoglobinemia >12,000 μg/L	Pulse steroid CYC	Resolved renal function
8	70/F	UTI	Moderna	3 weeks after 1st dose	Headache Hemoptysis Hematuria	Dysmorphic RBCUPCR:4,384 mg/g	SCr:3.5 mg/dL CRP:2.7 mg/dL	Anti-MPO (378 IU/ml)	Vasculitis with pauci-immune crescent GN	Pulmonary vasculitis	Plasma exchange Pulse steroid Rituximab	Improved renal function(SCr: 2.5 mg/dL)

*CYC, Cyclophosphamide, Hx, history, Bx, biopsy, NA, Not applicable, PR3, proteinase 3, ANCA, anti-neutrophil cytoplasmic antibody, HTN, hypertension, DM, diabetes mellitus, SCr, serum creatinine, GN, glomerulonephritis, UPCR, urine protein creatinine ratio, CKD, chronic kidney disease, CRP, C-Reactive protein, LLL, left lower lung*.

COVID-19 vaccination with different strategies and designs can trigger and enhance innate and adaptive immunity by activating neutrophils, T cells, and B cells (15). In contrast to ordinary inactivated viral or adjuvanted protein vaccines, the pioneer mRNA vaccination technique may trigger even stronger antigen-specific cluster of CD4+ and CD8+ T-cell responses and eventually exacerbate intensive immune-mediated diseases (16). Temporal AAV following COVID vaccination is reminiscent of the recently-reported AAV with immune-mediated glomerulonephritis or RPGN following COVID-19 infection in genetically susceptible patients, and may therefore share similar mechanisms of activation (17, 18). Possible mechanisms include polyclonal activation, molecular mimicry, and systemic proinflammatory cytokines response. The proinflammatory cytokines can prime neutrophils with further activation of neutrophil extracellular traps (NETs) formation and antibodies formation to myeloperoxidase and proteinase 3 (19, 20).

The wide-range timeline of AAV activation following COVID-19 vaccination has not been elucidated. AAV with RPGN after COVID-19 vaccination could be masked by initial non-specific prodromal symptoms, making early recognition difficult. For patients with the pre-existing asymptomatic or oligosymptomatic autoimmune diseases, COVID-19 vaccination may accelerate the immune activation more rapidly, compared to other *de novo* immune response. Due to the limited case number, clearer timeline of immune activation post COVID-19 vaccination requires large vaccination registration studies in the future.

Given the persistent COVID-19 pandemic and the emergence of variants of concern, the rare incidence of severe adverse events after COVID-19 vaccination should not be a reason for vaccine hesitancy. Nevertheless, our patient highlighted the potential development of AAV with RPGN and pulmonary involvement following COVID-19 vaccination, suggesting that the early examination of urinalysis and renal function is imperative for the prompt recognition of glomerulonephritis and acute renal failure in vulnerable patients. The potential causal relationship between COVID-19 vaccination and the development of *de novo* AAV or glomerulonephritis warrants further investigation.

## Data Availability Statement

The original contributions presented in the study are included in the article/supplementary material, further inquiries can be directed to the corresponding author/s.

## Ethics Statement

The studies involving human participants were reviewed and approved by Ethics Committee on Human Studies at Tri-Service General Hospital in Taiwan. The patients/participants provided their written informed consent to participate in this study. Written informed consent was obtained from the individual(s) for the publication of any potentially identifiable images or data included in this article.

## Author Contributions

C-CC authored the manuscript, with contribution from all authors. H-YC prepared the figure. C-CL was consulted for rheumatology problems. S-HL supervised the article. All authors contributed to the article and approved the submitted version.

## Conflict of Interest

The authors declare that the research was conducted in the absence of any commercial or financial relationships that could be construed as a potential conflict of interest.

## Publisher's Note

All claims expressed in this article are solely those of the authors and do not necessarily represent those of their affiliated organizations, or those of the publisher, the editors and the reviewers. Any product that may be evaluated in this article, or claim that may be made by its manufacturer, is not guaranteed or endorsed by the publisher.

## Abbreviations

AAV: anti-neutrophil cytoplasmic antibody-associated vasculitis
RPGN: rapidly progressive glomerulonephritis.
